# Genome-wide discovery, and computational and transcriptional characterization of an AIG gene family in the freshwater snail *Biomphalaria glabrata,* a vector for *Schistosoma mansoni*

**DOI:** 10.1186/s12864-020-6534-z

**Published:** 2020-03-02

**Authors:** Lijun Lu, Eric S. Loker, Si-Ming Zhang, Sarah K. Buddenborg, Lijing Bu

**Affiliations:** 1 Center for Evolutionary and Theoretical Immunology, Department of Biology, University of New Mexico, Albuquerque, NM 87131 USA; 2 Wellcome Sanger Institute, Wellcome Trust Genome Campus, Hinxton, CB10 1SA UK

**Keywords:** AIG gene, AIG1 domain, GIMAP, IAN, Coiled-coil, Conserved motif, *Biomphalaria glabrata*, *Schistosoma mansoni*, Mollusca, Invertebrate, Gene expression

## Abstract

**Background:**

The AIG (avrRpt2-induced gene) family of GTPases, characterized by the presence of a distinctive AIG1 domain, is mysterious in having a peculiar phylogenetic distribution, a predilection for undergoing expansion and loss, and an uncertain functional role, especially in invertebrates. AIGs are frequently represented as GIMAPs (GTPase of the immunity associated protein family), characterized by presence of the AIG1 domain along with coiled-coil domains. Here we provide an overview of the remarkably expanded AIG repertoire of the freshwater gastropod *Biomphalaria glabrata*, compare it with AIGs in other organisms, and detail patterns of expression in *B. glabrata* susceptible or resistant to infection with *Schistosoma mansoni*, responsible for the neglected tropical disease of intestinal schistosomiasis.

**Results:**

We define the 7 conserved motifs that comprise the AIG1 domain in *B. glabrata* and detail its association with at least 7 other domains, indicative of functional versatility of *B. glabrata* AIGs. AIG genes were usually found in tandem arrays in the *B. glabrata* genome, suggestive of an origin by segmental gene duplication. We found 91 genes with complete AIG1 domains, including 64 GIMAPs and 27 AIG genes without coiled-coils, more than known for any other organism except *Danio* (with > 100). We defined expression patterns of AIG genes in 12 different *B. glabrata* organs and characterized whole-body AIG responses to microbial PAMPs, and of schistosome-resistant or -susceptible strains of *B. glabrata* to *S. mansoni* exposure. *Biomphalaria glabrata* AIG genes clustered with expansions of AIG genes from other heterobranch gastropods yet showed unique lineage-specific subclusters. Other gastropods and bivalves had separate but also diverse expansions of AIG genes, whereas cephalopods seem to lack AIG genes.

**Conclusions:**

The AIG genes of *B. glabrata* exhibit expansion in both numbers and potential functions, differ markedly in expression between strains varying in susceptibility to schistosomes, and are responsive to immune challenge. These features provide strong impetus to further explore the functional role of AIG genes in the defense responses of *B. glabrata,* including to suppress or support the development of medically relevant *S. mansoni* parasites.

## Background

Characterization of the immune defense capabilities of invertebrates has been aided by the increasing number of available genomes, more comprehensive transcriptional studies that outline invertebrate responses to a variety of pathogens, and by the rapidly growing availability of bioinformatics tools to enable analysis and comparison of such responses [1–3]. Invertebrate defenses are complex and often involve deployment of unexpectedly large families of immune-related molecules [4–6]. In addition to large gene families the individual members of which might be expressed in specific ways following particular kinds of stimuli [5, 6], other mechanisms to diversify invertebrate responses such as allelic diversity, alternative splicing and somatic recombination have been reported, adding to the potential of invertebrates to fine-tune their responses to pathogens [7–10]. Additionally, different invertebrate groups may be challenged by distinctive kinds of infectious agents, as for example from particular groups of fungal or metazoan parasites with which they are regularly exploited and with which they have co-evolved [11]. Consequently, the overall array of invertebrate responses becomes very impressive.

The study of invertebrate immunity has been aided by investigations of the defense responses of plants and vertebrates, and vice versa [12]. One example of a group of immune-related molecules first discovered in plants and mammals is the AIG family of GTPases. The first family member, AIG1 (avrRpt2-induced gene), was discovered in *Arabidopsis thaliana* and its expression was induced by exposure to plant pathogens or abiotic stressors [13–15]. The AIG1 domain consists of G1 through G5 boxes and two unique conserved motifs, the consensus box CB located between G3 and G4, and the IAN (immune-associated nucleotide-binding protein) consensus sequence that partially overlaps the G5 region. The AIG1 domain comprises a GTP binding region, and genes containing an AIG1 domain are called AIG genes. In plants and vertebrates, the AIG family of GTPases is frequently represented by GIMAPs (GTPase of the immunity associated protein family), also known as immune-associated nucleotide binding proteins or IANs. GIMAPs are proteins of 30–80 kDa containing coiled-coil regions along with a characteristic AIG1 domain. Some GIMAP genes encode proteins with membrane-anchoring domains whereas others are soluble proteins [16].

In mammals where they have been most extensively studied, GIMAPs are involved in regulating and maintaining T cell numbers and survival [17, 18]. They are associated with both proliferative and apoptotic processes [16]. GIMAPs are also known from the coral *Acropora millepora* for which a role for GIMAPs in phagolysosomal processing was proposed [19]. In support of this idea, GIMAPs found in Lewis rats resistant to the apicomplexan parasite *Toxoplasma gondii* have been implicated in binding to the parasitophorous vacuole surrounding these intracellular parasites and favoring fusion with host cell lysosomes, thereby leading to the demise of the parasites [20]. GIMAPs may be linked to clinically relevant phenomena like T-cell leukopenia autoimmunity or leukemia.

One of the most interesting aspects of GIMAP biology is their peculiar phylogenetic distribution. They are found in plants [21], some protists like *Entamoeba* [22], corals but not all cnidarians [19], gastropod [23–25] and bivalve molluscs [5, 23, 26], in the cephalochordate *Branchiostoma* (lancets) and the hemichordate *Sacoglossus* (acorn worm) and in vertebrates [19, 23]. They are lacking as far as is known in representative fungi like yeast like *Saccharomyces cerevisae*, early diverging metazoans such as the placozoan *Trichoplax* or the sponge *Amphimedon*, or in the model ecdysozoans *Caenorhabditis elegans* or *Drosophila melanogaster*. A recent study predicted 60 AIG1 genes in the genome of the sub-terrestrial, thermally-stressed nematode *Halicephalobus mephisto* [27]. In deuterostomes, they are not known from the tunicate *Ciona*, the sea urchin *Strongylocentrotus* or from lampreys [19, 23].

With respect to numbers of GIMAP loci, *Arabidopsis* has 13 [28], the oyster *Crassostrea virginica* has 28 [23], and zebrafish have over 100 [19]. Rats have 7 genes, humans 8 and mice 9 [29, 30]. AIG gene family expansions have occurred in bivalves and in the nematode *H. mephisto* [19, 23, 27]. GIMAP loci are clustered in plants, corals and mammals, suggestive of tandem gene duplications [16, 23, 28, 29]. The phylogenetic distribution is consistent with an ancient origin for GIMAPs accompanied by independent losses in some lineages and amplifications in others [19, 23, 31]. A note of caution has been expressed that the similarities between plant IANs and animal GIMAPs may represent convergence [19].

Our interest in AIG genes is centered on planorbid gastropods in the genus *Biomphalaria*, particularly the Neotropical species *B. glabrata* and African species such as *B. pfeifferi*. These snails serve as vectors of the human parasite *Schistosoma mansoni*. Schistosomes are responsible for schistosomiasis, a neglected tropical disease that still infects over 200 million people [32, 33]. In a microarray-based study of the transcriptomic responses of the hematopoietic organ of *B. glabrata,* four GIMAPs were found to be significantly up-regulated following exposure to bacterial lipopolysaccharide (LPS) and peptidoglycan (PGN) [24]. An RNA-Seq study of the transcriptomic responses of *B. pfeifferi* to *S. mansoni* revealed that GIMAPs were up-regulated at both one and three days post-exposure [25]. Given the presence of GIMAPs in *B. glabrata*, their responsiveness to immune stimuli including schistosomes, and the association of GIMAPs with immune cell numbers and regulation noted in other model systems, we undertook a further examination of the AIG gene family in *B. glabrata*. Our studies are motivated by the need to develop novel methods of schistosome control based on development of snails resistant to schistosome infection.

## Results

The following definitions are used throughout the paper: **AIG1 domain**: a conserved domain including G1-G4, G5/IAN motifs and a conserved box (CB) found with the predicted protein sequences; **partial AIG gene:** gene containing an incomplete AIG1 domain, with absence of at least one conserved motif; **AIG gene:** gene containing at least one complete AIG1 domain, and possibly other domains; ***B. glabrata*** **GIMAP**: *B. glabrata* gene containing a complete AIG1 domain and one or more coiled-coil domains. We refer to the more formal names for designated genes like “BGLB008770” as “Bg8770” for the sake of readability. The “-PB” or “-RB” suffix following the gene ID is the protein ID or transcript ID of the corresponding gene, respectively.

### Conserved motifs within the *B. glabrata* AIG1 domain

**Fig. 1:**
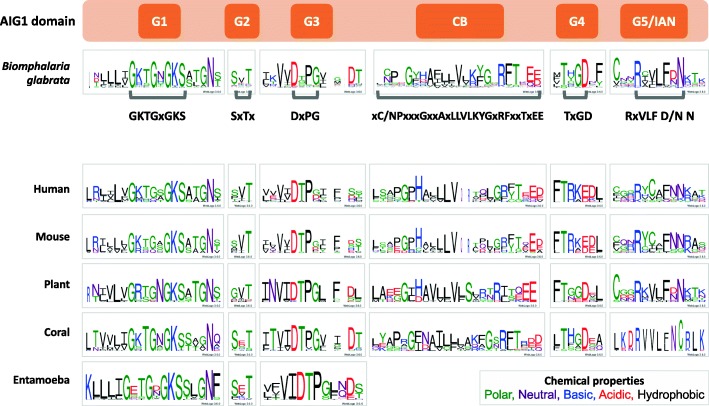
Conserved motifs within the *B. glabrata* AIG1 domain. Accurate locations of conserved AIG1 motifs (G1 = GKTGxGKS; G2 = SxTx; G3 = DxPG; CB = xC/NPxxxGxxAxLLVLKYGxRFxxTxEE; G4 = TxGD; G5/IAN = RxVLF D/N N) in *B. glabrata* were marked out with grey bars under each logo. Consensus sequences for AIG1 domains in other organisms were collected from published literature: human (*Homo sapiens*) [29], mouse (*Mus musculus*) [16], plant (*Arabidopsis thaliana*) [28], coral (*Acropora millepora*) [19], and entamoeba (*Entamoeba histolytica*) [22]

Conserved motifs (G1-G4, G5/IAN and CB) within the AIG1 domains of *B. glabrata* are indicated in Fig. 1, with more details in Additional file 2: Table S1, and occur in the order expected. In addition to consensus signatures, the motifs also contain some unique sequences as compared to known motif variants in AIG1 domains from other organisms. Motifs G1-G3 in *B. glabrata* are similar to those originally defined from human sequences. For example, protein sequences like **G**xxxx**GKS**, the conserved G1 motif in other organisms, can also be found near the beginning of the *B. glabrata* AIG1 domain sequence. Multiple sites of similar conservation within the G1 motif such as GKTGxGKS can also be found. Conserved sequences flanking the G1 motif in *B. glabrata* are also observed, such as LLLT/V on the N-terminal and A/STGNS/T on the C-terminal sides. Similarly, G2 and G3 motifs in *B. glabrata* are Sx**T**x and **D**xP**G** (Fig. 1). The CB, G4 and G5/IAN motifs in *B. glabrata* exhibit some variation compared to other organisms, but still can be accurately located by reference to known consensus sequences for the motifs, their relative location and their flanking conserved sequences. The CB consensus sequence in *B. glabrata* xC/N**P**xxx**G**xx**A**x**LLV**LKY**G**x**RF**xx**T**x**EE** (Fig. 1) has the most similarity to the mouse counterpart (LSx**PG**PH**A**L**LLV**xQL**G**-**RF**/Y **T**x**E D/E**), which can be found near the C-terminus of the G3 motif. The G4 motif in *B. glabrata* (**T**x**GD**) has consensus sequence variations of **NK**x**D** in human, mouse and plant, and **T**x**C**D/E in coral. The G5/IAN motif in *B. glabrata* has a **R**xVL**F** D/**N N** signature, which is partially overlapping with the mouse IAN motif **R**xxx**FNN** K/R AxxxE. In the mouse AIG1 domain, the G5 motif is embedded in IAN (xxx in **R**xxx**FNN**), while the corresponding location in *B. glabrata* contains a less conservative sequence (amino acids of top frequencies are C28%, V60%, L86%), resulting in “xVL” in **R**xVL**F** D/**N N**).

### Overview of the *B. glabrata* AIG gene family

An initial scan with the HMM AIG1 domain profile (PFAM ID: PF04548) returned genes containing AIG1 domains as well as domains belonging to other GTPase families within the same P-loop NTPase superfamily. All non-AIG1 GTPase genes were filtered out after scanning with the InterProScan profile. In total we found 111 genes (148 predicted proteins) with complete or partial AIG1 domains (Additional file 3: Table S2). Of these, 91 genes (128 proteins) had complete AIG1 domains, and the remainder had AIG1 domains missing G1, G5/IAN or additional motifs and were considered to be partial AIGs.

**Fig. 2:**
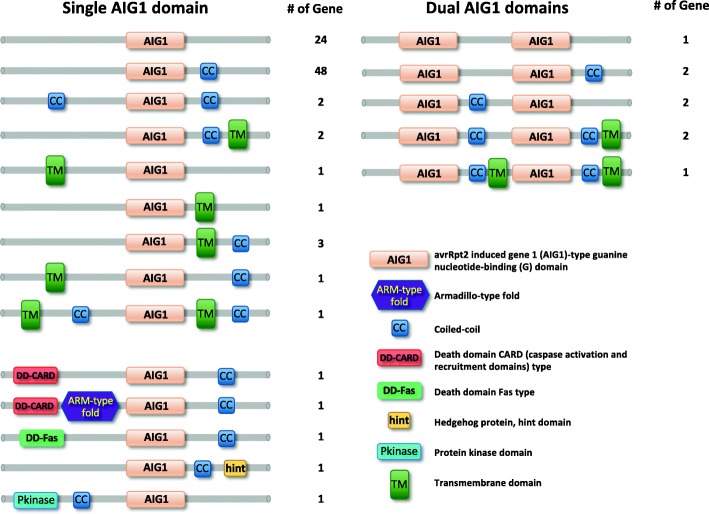
Predicted domain architectures of AIG genes in *B. glabrata*. Conserved domains were predicted using InterProScan which collected protein signatures from 14 specialized databases. The coiled-coil (CC) icon may indicate from 1 to 4 tandem coils and the TM icon from 1 to 3 transmembrane domains

The 91 complete AIG genes exhibit 19 different arrangements of domain architectures based on predictions using InterProScan (Fig. 2). Taking into account alternative splicing, an additional five domain architectures were found. For example, gene *Bg8770* has three splice variants: DD-CARD + AIG1 + coiled-coil (Bg8770-RB), AIG1 + coiled-coil (Bg8770-RC) and DD-CARD + ARM-type-fold + AIG1 + coiled-coil (Bg8770-RD).

There are 64 genes (101 proteins) with an AIG1 domain and at least one predicted coiled-coil, meaning they fit the criteria associated with a *B. glabrata* GIMAP. For the following analyses we also considered the 27 AIG-containing genes without coiled-coils because: a) the evolutionary history of GIMAP genes is likely to be entwined with AIG genes lacking coiled-coils; and b) coiled-coil domains can be missed by prediction tools because they vary considerably in length (87~3000 aa) and degree of sequence conservation (from 29.4% to hypervariable 97.1%) [34]. Coiled-coil domains could be identified on either side of AIG1 domains, indicating the possibility of polymerization, possibly influencing ligand binding [16, 35]. Detailed predictions for classification as antiparallel and parallel dimers, trimers and tetramers based on LOGICOIL are listed in Additional file 3: Table S2.

We also found genes containing unusual or complex domain architectures including dual AIG1 domains, an AIG1 domain with additional N-terminal death domains (DD) or protein kinase (Pkinase) domains, with an N-terminal Armadillo (ARM)-type fold, or a C-terminal hint/hedgehog domain (Fig. 2). Because of variable domain architectures, AIG proteins were predicted to range from 105 aa (19-kDa) for incomplete AIG1 domains to 1286 aa (141-kDa).

### Transmembrane predictions for the AIG family

**Fig. 3:**
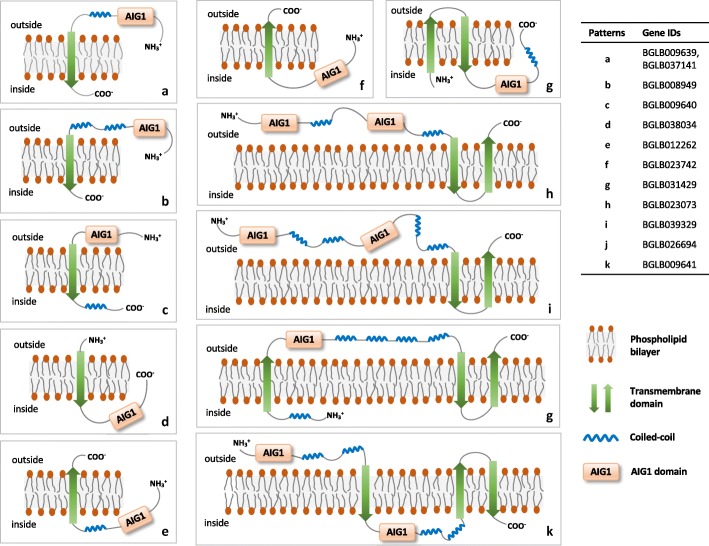
Hypothetical transmembrane (TM) dispositions of predicted polypeptides of the AIG genes of *B. glabrata*. The phospholipid bilayers here could represent not only plasma membranes, but also could be membranes of organelles including mitochondria, endoplasmic reticulum (ER), Golgi apparatus or other membranous organelles. There are 3 types of hypothetical TM dispositions: Type I, a single transmembrane span with N-terminus on the outside (**a**-**d**); Type II, a single transmembrane span, with C-terminus on the outside (**e**-**f**); and Type III, with multiple spans (**g**-**k**). Some models predict the presence of an AIG1 domain on both side of a membrane

No signal peptide was encoded by any of the AIG genes. Additionally, 12 AIG genes contained predicted transmembrane domains (TM), suggestive of their location on the plasma membrane or intracellular membranes. A further look based on TMHMM results revealed interesting differences in membrane spanning structures (Fig. 3). TM domain numbers ranged from 1 to 3, with at least three types of structures noted. Additionally, the AIG1 domain can be on either side of the membrane, adding an extra layer of potential functionality. Lastly, 10 out of 12 TM domain-containing AIG genes are associated with coiled-coils, indicating the possibility of multimerization on the membrane.

### Scaffold locations and arrangements of AIG genes in *B. glabrata*

**Fig. 4:**
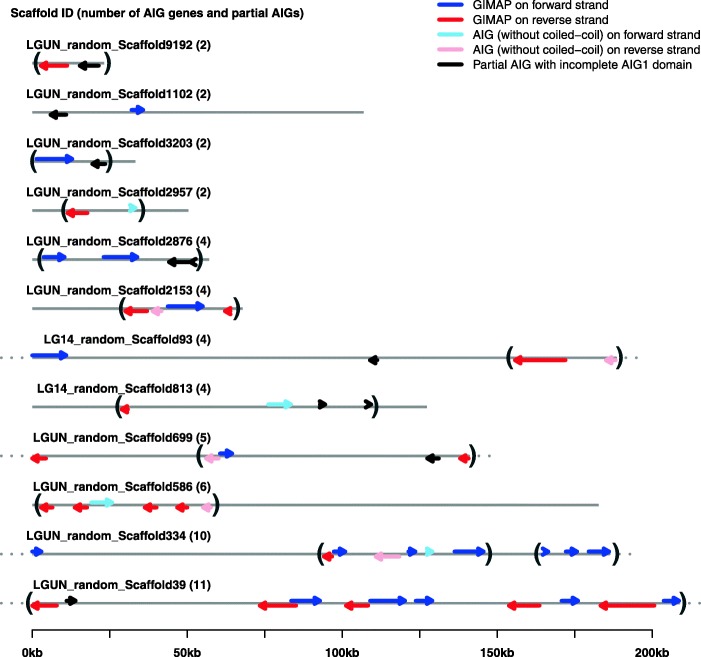
Scaffold locations of evolutionary footprints of AIG genes in the *B. glabrata* BB02 genome. The evolutionary footprints of AIG genes in *B. glabrata* consist of three types: GIMAP (AIG gene with coiled-coil domain), AIG gene without coiled-coil domain, and partial AIGs. Scaffold backbones were drawn with gray lines. Scaffolds longer than the figure region were marked with gray dots on left or right end of the gray lines. Genes on the forward strand were marked out using left-to-right arrows above scaffold lines, showing GIMAP genes (blue) and AIG genes (sky blue). Genes on the reverse strand were marked out using right-to-left arrows below scaffold lines, showing GIMAP genes (red) and AIG genes (pink). Partial AIGs (black) were showing on both forward and reverse strands. Scaffold IDs were labeled above each scaffold. Numbers in parenthesis after scaffold IDs are total number of AIG genes (with and without coiled-coils) on the scaffold. Gray parentheses enclosed genes within the same tandem array (no other genes in between)

We included both complete and partial AIG genes in this analysis. The reference genome of *B. glabrata* BB02 strain contains 331,400 scaffolds, 13,826 of which have been annotated. The AIG footprints are located on 66 different scaffolds (Additional file 1: Fig. S1), thirteen of which contain at least two complete or partial AIG genes. Tandem arrays of complete or partial AIGs were found on 12 scaffolds (Fig. 4). For example, on Scaffold 39, there are 11 AIG genes forming one tandem array, 10 of which are *B. glabrata* GIMAPs. Similarly, on Scaffold 334, there are two tandem arrays with 9 AIG genes, 8 of which are GIMAPs. A total of 50 AIG footprints (31 GIMAPs, 9 AIG genes without coiled-coils, and 10 partial AIGs) were found to in tandem arrays ranging 2 to 11 genes. There are three orientation types among the tandem gene pairs: 1) parallel → → or ← ← (16 pairs); 2) convergent → ← (10 pairs); and 3) divergent ← → (12 pairs). In Fig. 4, the gray brackets on some scaffolds show the AIG genes in tandem array. Additionally, 55 genes are dispersed on the other 54 scaffolds. For example, Scaffold 43 contains two GIAMPs separated by 400 kb (Additional file 3: Table S2, Additional file 1: Fig. S1).

### AIG gene expression analysis in *Biomphalaria spp*

#### I). Constitutive expression in different snail organs [36]

**Fig. 5:**
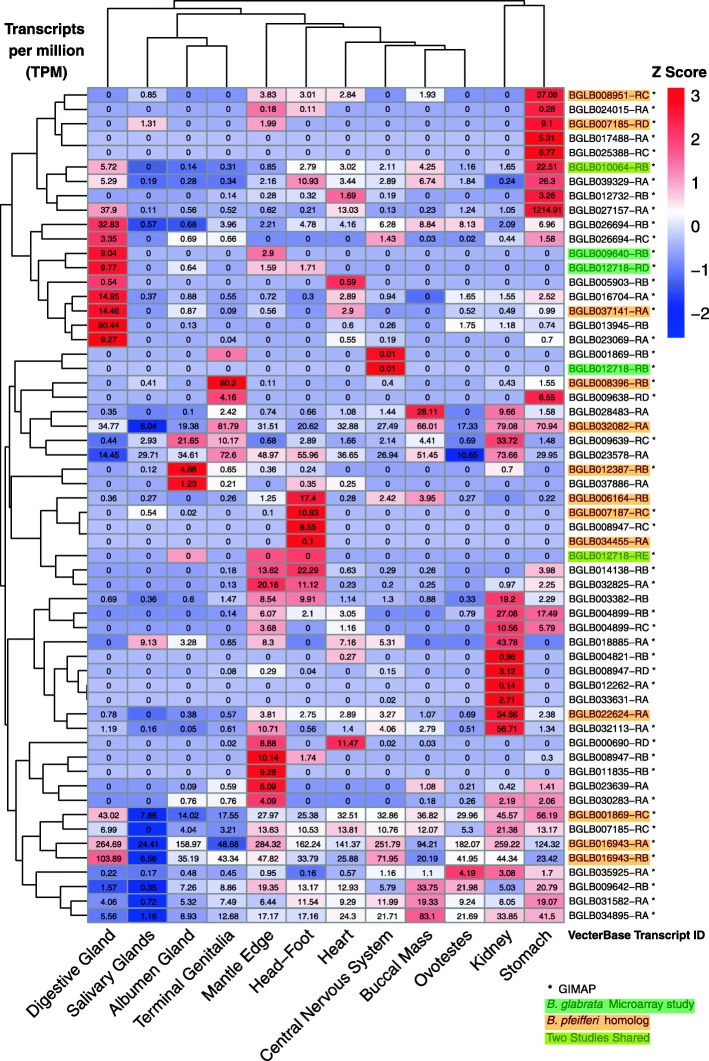
Heatmap of organ specific expression of *B. glabrata* AIG genes. RNA-Seq reads from 12 organs of *B. glabrata* BB02 [36] were analyzed. Blocks in the heat map were colored in reference to Z scores transformed from transcripts per million (TPM). Z scores were calculated as (TPM – mean across organ)/standard deviation across organs. The Z score is a cross-organ normalization of TPM for each individual gene, and for a given gene, Z scores among organs are comparable. To compare gene expression within one specific organ, TPM was used. VectorBase transcript IDs were used to match with a specific AIG gene on each row. Those IDs with an asterisk are *B. glabrata* GIMAPs. IDs in green were target sequences of microarray probes in the study on gene expression of *B. glabrata* BS-90 strain (schistosome-resistant) injected with pathogens [24]. IDs in orange were homologs of AIG genes (cut-off values: > 70% identity; > 90% coverages) for a related snail, *B. pfeifferi,* from RNA-Seq data [25]. IDs in mustard color were AIG genes that appeared in both studies above

Organ specific gene expression was assessed using RNA-Seq data from 12 organs of unstimulated *B. glabrata* BB02 strain snails [36]: buccal mass, kidney, heart, central nervous system, digestive gland, ovotestes, stomach, albumen gland, terminal genitalia, head foot, mantle edge, and salivary gland. Based on transcripts per million (TPM) transformed Z scores, 47 GIMAPs and 11 additional AIG genes showed significant gene expression (Fig. 5, and Additional file 5: Table S4 for domain and other features). The most highly expressed transcripts were found in stomach, digestive gland and terminal genitalia. Each organ had a specific pattern of AIG gene expression, but some pairs of organs were more similar to each other than to others (e.g. stomach and kidney, or albumen gland and terminal genitalia). There were also several “clusters” of transcripts with similar expression patterns among the organs. In digestive gland, 10 transcripts (encoded by 1 AIG and 8 GIMAP genes) were preferentially expressed and clustered together, four of them with transmembrane domains. In stomach, 9 GIMAP transcripts originating from widely distributed scaffolds were overexpressed, of which two have dual AIG1 domains and one a transmembrane domain. In the salivary glands, 8 transcripts (encoded by 7 GIMAPs) were under-expressed, one of which has hint/hedgehog domains.

#### II). Analysis of AIG gene expression from previously published microarray study of *B. glabrata:* responses to immunogens LPS (lipopolysaccharide), PGN (peptidoglycan) or FCN (fucoidan) [24]

Gene expression values (Fig. 5, Additional file 5: Table S4) of the schistosome resistant BS-90 strain of *B. glabrata* indicated that four GIMAP genes (Bg9640, Bg11834, Bg25758 and Bg21576) were significantly up-regulated (from 3- to 13-fold) following injection with LPS [24]. The first two are categorized as *B. glabrata* GIMAPs, with Bg9640 having a transmembrane domain (see also Fig. 3b). Bg9640 was also highly expressed in digestive gland and mantle edge (Fig. 5). The third gene Bg25758 identified by Zhang et al. [24] lacked G1-G3 motifs and coiled-coils. Their fourth gene originally annotated as a GIMAP we reannotated as “non-coding RNA” based on NCBI gene bank (XR_001217856.1) and VectorBase v1.6 entries.

We also discovered 10 more AIG genes match probes on the DE genes list in the microarray study (Fig. 5, Additional file 5: Table S4). All 10 genes were initially annotated as “NA” (no Genbank match) but based on our AIG criteria they include 7 GIMAPs, 2 complete AIG, and 1 partial AIG (Additional file 5: Table S4). One of the GIMAP genes (Bg17413) was significantly up-regulated in snails exposed to LPS (18.6 fold) or to PGN (5.7 fold). Conversely, the GIMAP gene Bg10064 was down-regulated 2.4 fold following FCN treatment. FCN is a complex polysaccharide derived from the brown alga *Fucus vesiculosus* and is thought to mimic fucosyl-rich glycans found on the surfaces of sporocysts of *S. mansoni* [24]**.**

#### III). AIG analysis from previously published RNA-Seq results obtained for field-derived specimens of the African schistosome vector snail *Biomphalaria pfeifferi* exposed to *S. mansoni* [25]

*Biomphalaria pfeifferi* is a widely distributed and important vector for *S. mansoni* in sub-Saharan Africa, and is a close relative of *B. glabrata*. A total of 28 *B. pfeifferi* homologs for *B. glabrata* AIG genes were identified using a BLASTp search cutoff of 70% identity and 90% alignment coverages on both query (translated *B. pfeifferi* transcripts) and subject (*B. glabrata* BB02 annotated proteins). Of these, 19 were GIMAPs. DE analysis of *B. pfeifferi* AIG genes (Additional file 5: Table S4) show that at 3-day post-exposure (dpe) to *S. mansoni*, 4 GIMAPs were significantly up-regulated (from 4.66- to 40.20-fold) and 1 GIMAP gene was significantly down-regulated (2.32-fold). With the cutoff values for DE genes used in this study (PPDE ≥ 0.95, and fold change > 2), no AIG genes were detected to be significantly expressed at 1 dpe to *S. mansoni* or in shedding snails (Additional file 5: Table S4). Additionally, 15 *B. glabrata* AIG genes with clear *B. pfeifferi* homologs were identified in the organ expression study in Fig. 5.

#### IV). Comparisons of expression levels of AIG gene family members in strains of *B. glabrata* susceptible (M line) or resistant (BS-90) to *S. mansoni*, before and after exposure to this parasite

At constitutive levels (Table 1, Additional file 4: Table S3, Additional file 5: Table S4), for at least one of the three direct strain comparisons, 34 AIG genes were more highly expressed in schistosome-resistant BS-90 snails than in susceptible M line snails (19 GIMAPs, 11 AIG genes without coiled-coils and 4 partial AIG genes). Several AIG family members showing no expression in resting M line snails were notable for being expressed at much higher resting levels in BS-90 snails. Among them, Bg17413 (GIMAP) and Bg34408 (AIG) have exceptionally higher average raw reads counts (~ 3000) compared to the rest 32 AIG genes (~ 300). Conversely, 21 AIG genes were over-expressed in M line relative to BS-90 snails (13 GIMAPs, 5 AIG without coiled-coils and 3 partial AIG genes).

**Table 1:**
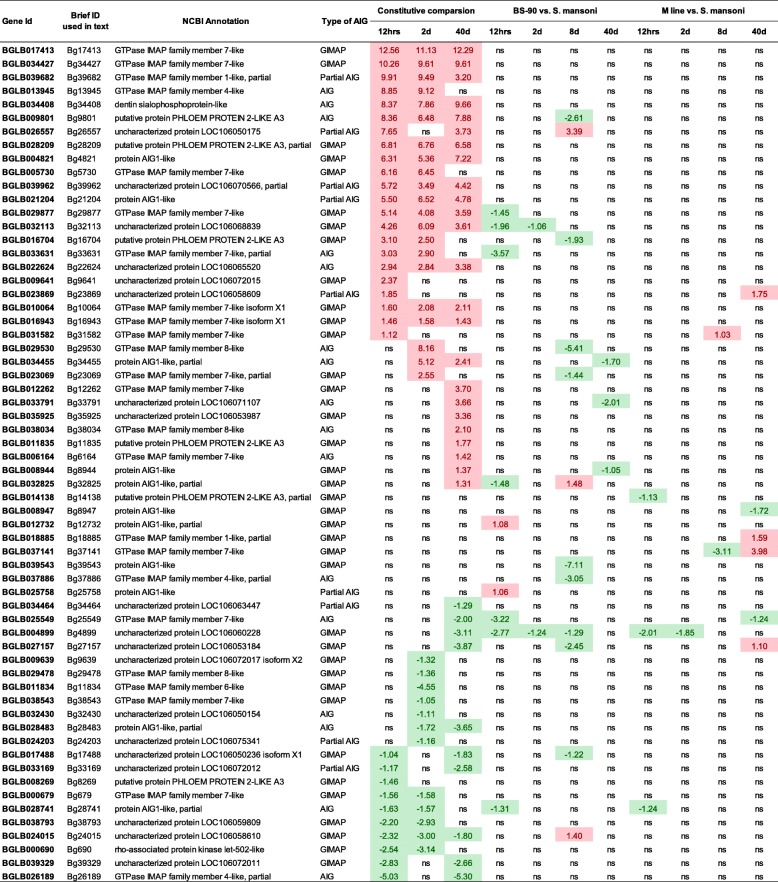
Summary of AIG genes expression in *B. glabrata* BS-90 and M line at constitutive level and response to *S. mansoni* exposure

Notes

1. Value in cells represents the value of log2(fold change) of the gene. Any up-regualted DE gene with log2(fold change) greater than 1 is highlighted in red, and any down-regualted DE gene with log2(fold change) less than -1 is highlighted in green

2. The **12 h**, **2d**, **8d** and **40d** represent the 4 sampling time points, which are time-matched unexposed controls and exposed snails for comparison

3. ns: no significant expression difference detected. Gene with PPDE> 0.95 and fold change > 2 is considered to be differentially expressed in this study

Shown are 3 comparisons of constitutive levels of AIG gene expression in BS-90 snails relative to M line snails (all snails were unexposed time-matched controls, with those at 0.5 and 2 dpe representing juvenile snails and those at 40 dpe being adult snails). Also, for both M line and BS-90 snails, there are 4 separate within strain comparisons of the effects of S. mansoni exposure (for 0.5, 2, 8 and 40 dpe) on AIG gene expression. Note that by 40 dpe, exposed BS-90 snails contain little or no S. mansoni DNA, and exposed M line snails were shedding cercariae

Both *B. glabrata* strains showed relatively modest differences in AIG gene expression between unexposed and *S. mansoni*-exposed snails. For both strains the overall, but by no means exclusive trend, was towards down-regulation in snails exposed to *S. mansoni*. Bg7141 was notable for being upregulated in M line snails shedding *S. mansoni* cercariae. Many (49) of the 111 AIG genes (including partial AIG genes) showed no transcriptional differences (fold change ≤2) between strains, or within a strain following exposure to infection. GIMAPs comprised the majority (37 of 62) of all significantly expressed AIG genes in this study. Regarding special domains, only the Pkinase domain (Bg690) or dual-AIG1 domains were represented as significantly expressed among strain comparisons (Additional file 5: Table S4).

### Analysis of relationships among *B. glabrata* AIG genes

**Fig. 6:**
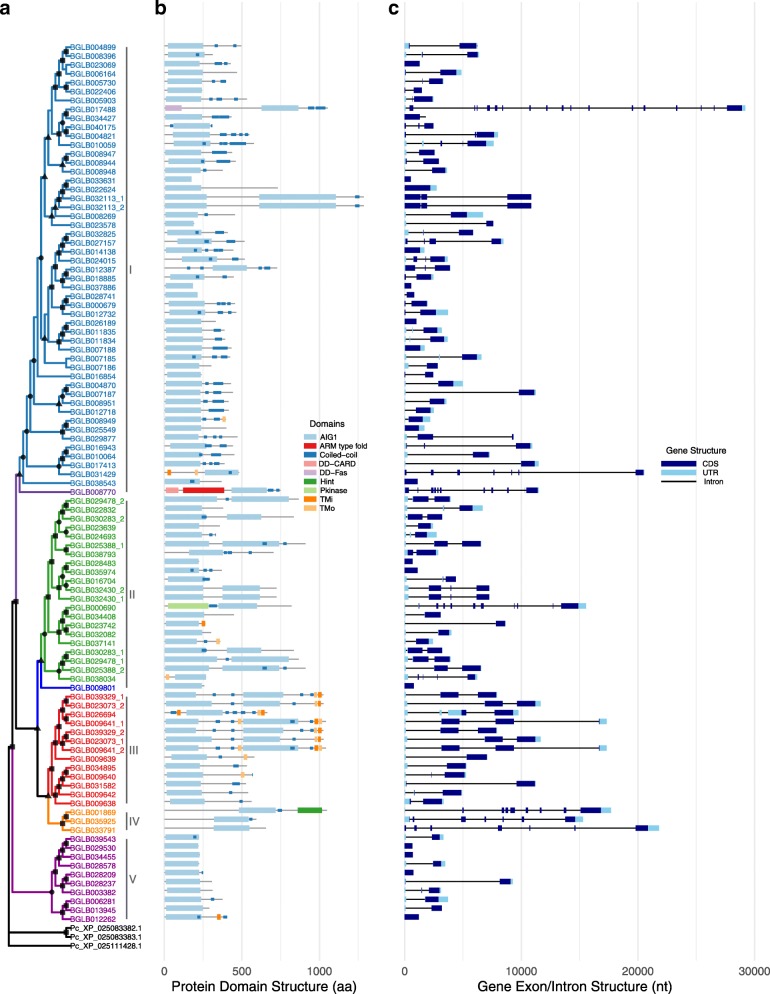
Relationships, domain architecture and gene structure of AIG genes in *B. glabrata*. **a.** A maximum likelihood (ML) phylogenetic tree of AIG1 domain sequences, with bootstrap values of 50 and greater noted with black shapes on the nodes (square 90–100, circle 70–89, triangle 50–69). For those AIG genes with dual-AIG1 domains, labels of “_1” and “_2” were added to the corresponding gene ID to differentiate the first and the second AIG1 domains. The tree topology revealed five clades (I-V) with well-supported bootstrap values (> 75), with two separate individual branches appearing among the five clades. **b.** Domain architectures of representative protein sequence of each AIG gene are listed. The featured domains include AIG1, coiled-coil, TMi (transmembrane domain from inside to outside of a membrane), TMo (transmembrane domain from outside to inside of a membrane), and other special functional domains: DD-CARD (death domain CARD type), DD-Fas (death domain Fas type), Hint (hint/hedgehog), armadillo (ARM)-type fold and Pkinase (protein kinase). **c**. Gene structures corresponding to representative transcripts of AIG genes were shown with exon, intron and untranslated region (UTR) in different colors

Shown in Fig. 6a is a tree of 91 AIG genes with complete AIG1 domain sequences for *B. glabrata* (sequence alignments in Additional file 6: File S1). This tree revealed five different clades (I-V) of *B. glabrata* AIG1 domain sequences, with good bootstrap support values (> 75%). There are two individual branches representing the genes Bg8770 and Bg9801 with slightly lower bootstrap support (both at 68%).

In Fig. 6b, a schematic of each AIG gene is presented showing the different types of domains represented with different colors. The majority of the 91 AIG genes contain one AIG1 domain, but there are 8 AIG genes with dual AIG1 domains distributed in clades I, II and III. Both AIG1 domains were included within the gene tree analysis. When two AIG1 domains occur in the same gene, they tend to cluster within the same clade (for example, Bg32113_2 clusters with Bg32113_1 in clade I), although such pairs are not always each other’s closest match. The other AIG genes containing special functional domains (death domains, protein kinase domain, hint/hedgehog domain, armadillo-type fold) were dispersed on the tree.

We noted AIG genes that lie close together in the genome and have similar domain architectures tend to cluster together on the tree. For instance, for one group of AIG genes in clade III (Fig. 6a), all are located in one tandem array (Fig. 4), and all are GIAMPs. Three genes in one sub-cluster of this clade (Bg9641, Bg39329, and Bg23073) had dual AIG1 domains as well as transmembrane domains and coiled-coil domains. By virtue of containing the largest tandem array of AIG genes (10 GIMAPs and 1 partial AIG) with close evolutionary relationships and by having dual-AIG1 domains, one particular scaffold region Scaffold 39:335311–543,941 seems to be a potential evolutionarily hot spot for generation of GIMAP genes through tandem gene duplication.

Gene structures of the 91 AIG genes were summarized with exon, intron and untranslated region (UTR) in different colors (Fig. 6c). Most of the gene structures show that one exon encodes one AIG1 domain and dual-AIG1 domains were encoded by two exons.

### AIG genes in 9 additional species of Mollusca

The same workflow used to identify *B. glabrata* AIG genes was applied to 9 other molluscan species with well-annotated genomes (Table 2). Although the initial AIG1 domain search with the HMM profile revealed some possible relevant hits, the follow up InterProScan analysis showed that some of these candidates actually belonged to other GTPase families with the same superfamily P-loop NTPase structure. For example, initially, 20 putative candidate genes were discovered with the HMM profile in *Octopus bimaculoides* and 17 in *Euprymna scolopes*, however none were confirmed as bonafide AIG genes after screening by InterProScan. Here, only those fitting the AIG gene criteria of this paper were summarized in Table 2.

**Table 2:**
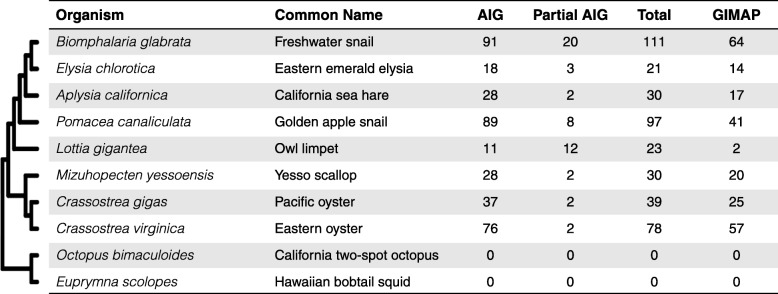
Summary of predicted numbers of AIG family members in *B. glabrata* and 9 molluscan species

Phylogenetic relationships among *B. glabrata* and 9 additional molluscan species were inferred according to published literature [37–39]

**Fig. 7:**
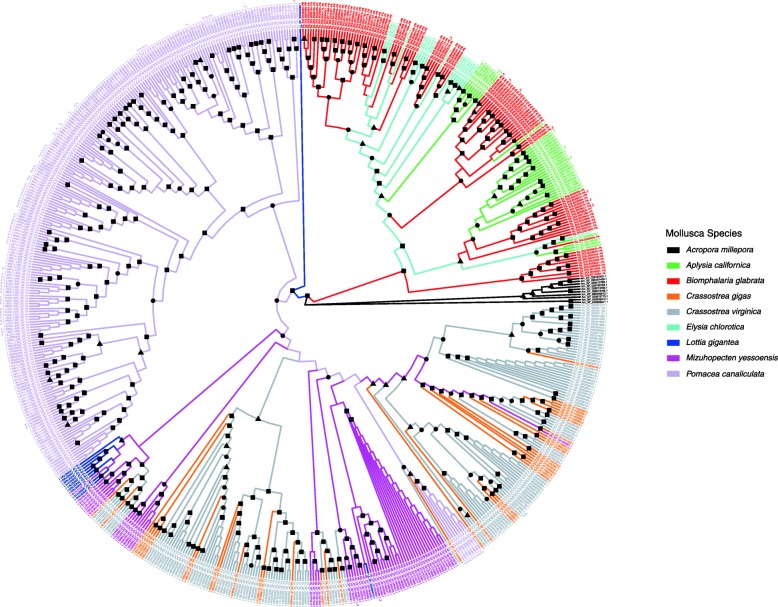
AIG gene tree for 8 molluscan organisms with complete genome annotation. The tree was built with AIG1 domains containing complete conserved motifs using IQ-TREE with standard model selection followed by tree inference of 1000 bootstrap search replicates (sequence alignments in Additional file 6: File S1). Also shown in black for reference are the AIG1 domain sequences from the coral *Acropora millepora* [19]. Bootstrap values of 50 and greater were indicated by shapes on the nodes (square 90–100, circle 70–89, triangle 50–69)

A maximum likelihood tree (Fig. 7, Additional file 6: File S1 for alignments) was constructed using complete AIG1 sequences from the 8 molluscan species with AIG genes from the coral *Acropora millepora* as outgroup. It is noteworthy that coral, gastropod and bivalve sequences were for the most part segregated from one another in the tree. One exception was the basal patellogastropod gastropod *Lottia gigantea* which had an isolated branch clustering within a number of sequences from the bivalve *Mizuhopectin*, and a small group of sequences clustering with another group of *Mizuhopectin* sequences (a small blue cluster at the bottom left, Fig. 7). Also noteworthy in this regard was *Pomacea* for which AIG1 domain sequences were diverse. Most arose from a single basal sequence distinct from the one basal sequence for the entire heterobranch group. However, a small subgroup of *Pomacea canaliculata* was also noticed embedded within a group otherwise composed of bivalve sequences. Interestingly, all members of this unusual subgroup are from the second, C-terminal AIG1 domain in dual-AIG1 domain containing genes. Heterobranch sequences were also diverse and showed evidence of distinct expansions among *Aplysia*, *Elysia* and *Biomphalaria*. In general, lineage specific expansions are observed as a major trend of molluscan AIG gene diversification with at least 3 such expansions noted for *Elysia*, 4 for *Aplysia* and 6 for *Biomphalaria*. Similar expansions are noted for the bivalves *Mizuhopectin* and the two *Crassostrea* species. As many as three lineage expansions can be traced back to a common basal state.

**Fig. 8:**
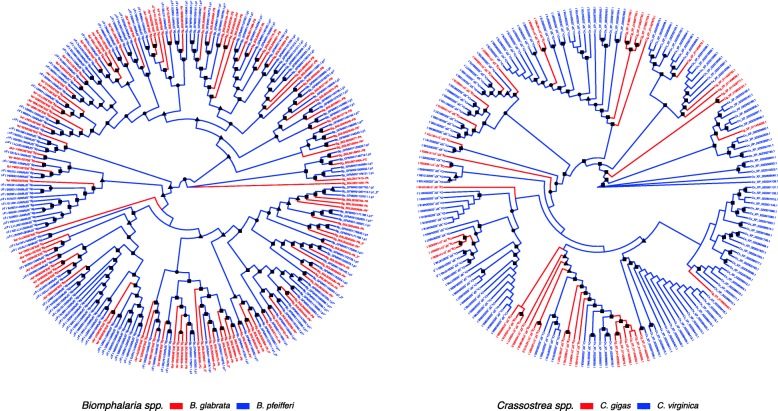
AIG gene tree for congeners *B. glabrata* and *B. pfeifferi* or *C. gigas* and *C. virginica*. The phylogenetic tree was built with AIG1 domains containing complete conserved motifs, using IQ-TREE with standard model selection followed by tree inference of 1000 bootstrap search replicates (sequence alignments in Additional file 6: File S1). Bootstrap values of 50 and greater were indicated by shapes on the nodes (square 90–100, circle 70–89, triangle 50–69)

We also examined AIG genes in a second prominent *Biomphalaria* species, the widespread African schistosome vector species *B. pfeifferi*. Although the whole genome of *B. pfeifferi* is not available, assembled transcriptome sequences generated from a previous RNA-Seq study were used [25]. We followed the same methods of AIG gene identification and gene tree construction (Fig. 8). The relatively close degree of relationship between the two taxa shown in this figure relative to Fig. 7 is revealed by the extent to which the AIG1 domain sequences of *B. glabrata* and *B. pfeifferi* are intermingled throughout the tree. Although a couple of early diverging single branches for *B. glabrata* were noted, the remaining groups initiated from deeper branches which seemed to originate with *B. pfeifferi* sequences, but most contained terminal branches from both species, again emphasizing the overall similar representation of AIGs across the two species. At least five major clusters contained only *B. pfeifferi* sequences, and the overall number of terminal branches represented by *B. pfeifferi* was higher (142) than for *B. glabrata* (101). As a comparison, an oyster AIG gene tree for the congeners *C. gigas* and *C. virginica* (Fig. 8, right) was also generated showing a clade distribution consistent with that seen in Fig. 7. More multi-member clades with species specific expansion were shown on the *Crassostrea* tree as compared with the *Biomphalaria* tree (Fig. 8, left).

## Discussion

The AIG gene family (containing GIMAPs) is recognized for its role in plants in defense from bacterial infection, and in mammals where GIMAPs play a role in regulation and maintenance of lymphocyte numbers and in phagolysosomal processing. GIMAPs are transcribed following injection of freshwater snail *Biomphalaria glabrata* with PAMPs like LPS [24]. Here for the first time we characterize the AIG gene family in *B. glabrata* which is an important vector for the widespread parasite of humans, *Schistosoma mansoni*. We also provide additional AIG family information for the related schistosome vector species, *B. pfeifferi*. We begin the exploration of their functional roles by documenting their expression patterns in various contexts including in *B. glabrata* strains susceptible or resistant to *S. mansoni,* and seek to explain origins and diversification of AIG genes of *B. glabrata* by comparison with those of other molluscs.

We first identified the essential motifs associated with the AIG1 domain in several *B. glabrata* AIG genes. Conserved motifs G1-G3, CB (conserved box), G4, G5/IAN were observed to have kept a consistent order and retained recognizable consensus sequences. We also observed distinct sequence substitutions within motifs unique to different species, including for *B. glabrata*. This is one potential indication of an ancient origin of the AIG1 domain, followed by substantial modifications in those groups of animals that have maintained the domain.

A total of 111 genes with complete or partial AIG1 domains were found in *B. glabrata*, of which 91 had complete AIG1 domains. The 91 genes with complete AIG1 domains were diverse with respect to the additional domains they contained, with 19 domain arrangements noted, five of which had two separated AIG1 domains. The functionality of *B. glabrata* AIG genes is potentially expanded by recruiting coiled-coils, transmembrane domains and other domains with specific functions. A multimerization option favored by the presence of coiled-coil domains may lead to further functional options involving larger macromolecular complexes [34]. The lack of signal peptides and the presence of transmembrane domains frequently found in *B. glabrata* AIGs are indicative of membrane-associated activities. Other specialized domains associated with the *B. glabrata* AIG1 domain include death domain superfamily members [40, 41], caspase activation and recruitment (CARD) domains [42, 43], and a protein kinase domain for a *B. glabrata* GIMAP (the latter also known from Atlantic salmon (*Salmo salar*) or fungi). Also found were Armadillo-type fold domains possibly facilitating large molecule (protein or nucleic acids) binding capability [44, 45] and a Hint domain associated with proteolysis functions [46].

An analysis of the distribution of AIG genes among the scaffolds of the BB02 *B. glabrata* genome shows that 50 AIG footprints (31 *B. glabrata* GIMAPs, 9 AIG genes without coiled-coils, and 10 partial AIG genes) were found to be tandemly arrayed in clusters ranging from 2 up to 11 genes. AIG genes with similar genomic location and functional features tend to cluster together. Although it is hard to reconstruct the mechanisms involved in AIG gene family expansions, the presence of AIG clusters and of occasional dual AIG1 domains in *B. glabrata* suggest segmental duplication as the main mechanism of family expansion. Clusters of tandemly arrayed AIG genes have also been found in humans and plants. Fusing of one open reading frame to another helps maintain a consistent ratio of transcription and translation for the co-occurring functional domains. Scaffold 39:335311–543,941, containing 10 evolutionarily similar GIMAPs in tandem array, seems like a potential hot spot for generation of GIMAP genes via tandem gene duplication. Tandem duplications (or equivalently unequal crossovers) have been found to account for 14% of all genes in 11 vertebrate genomes [47]. The high degree of homology seen in tandem arrays facilitates both unequal recombination and gene conversion which further reinforces homology resulting in large gene families [48]. Multiple rounds of unequal crossovers may lead to increases or decreases in the copy number of AIG genes, making it more flexible in response to changing environmental challenges [47, 48].

Towards a better functional understanding of AIG genes, we examined their expression in four different contexts (Additional file 5: Table S4). First, we profiled AIG genes expression in 12 different organs from unstimulated BB02 *B. glabrata.* We found over half of the AIG genes displayed considerable differences in organ-specific expression profiles, with some organs like the terminal genitalia and albumen gland having AIG gene expression patterns more similar to one another. More than 80% (47/58) of the AIG genes we noted to be expressed in the sampled organs are GIMAPs. Some groups of transcripts with similar patterns of expression were also noted. There is also evidence that these groups represent either related genes, or genes found within the same tandem array. For example, in the tandem array on the scaffold 39, two loci (Bg26694 and Bg9640) were both overexpressed in digestive gland, and three loci (Bg9642, Bg31582 and Bg34895) were all under expressed in salivary glands.

Second, we reexamined the expression results from the microarray study of schistosome resistant BS-90 *B. glabrata* injected with various PAMPs, including LPS [24]. Four AIG genes, two of which were GIMAPs, were among the most highly expressed genes recovered (up to 13-fold), strongly suggestive of a role for AIGs in response to bacterial PAMPs. Third, we also analyzed an extensive RNA Seq transcriptomics data for field-derived Kenyan *Biomphalaria pfeifferi* exposed for 1, 3, or 30+ days to *S. mansoni,* relative to unexposed controls [25]. Of 28 homologs for *B. glabrata* AIG genes identified in the *B. pfeifferi* dataset, four GIMAPs were highly expressed in schistosome-exposed snails at 3 days post-exposure, relative to unexposed control snails. Although the responses were not as dramatic as noted following injection of LPS, they nonetheless indicate snails normally highly susceptible to *S. mansoni* infection can mount an AIG response involving multiple genes.

Fourth, an RNA Seq transcriptomics study revealed that several AIG family members had much higher constitutive levels of expression (10 + fold) in the *S. mansoni*-resistant BS-90 snails relative to susceptible M line snails. Although neither snail strain showed particularly noteworthy or consistent changes in AIG gene expression following exposure to *S. mansoni* infection, the strikingly high constitutive expression shown by several AIG genes in BS-90 snails makes them excellent candidates as resistance-associated genes. One of these genes, Bg17413, a GIMAP gene (Additional file 5: Table S4), was significantly up-regulated in both *B. glabrata* exposed to LPS and PGN [24] and in *B. pfeifferi* snails at 3-day post exposure to *S. mansoni* [25].

Collectively the transcription studies reveal that AIG family members have complex patterns of expression in different organs, can respond strongly to bacterial PAMPs, and show remarkably high resting levels of expression in snails resistant to schistosomes. Further studies assessing the impact of knock-down of single or multiple highly expressed AIG genes on the resistance of BS-90 snails to *S. mansoni* should be undertaken. BG7141, a GIMAP highly upregulated in M line snails with full-blown patent infections of *S. mansoni* should also be investigated as a possible growth factor for *S. mansoni* or as part of a response to protect the snail from consequences of infection. Other AIG genes, such as those responding most dramatically to PAMP exposure (Bg11834, Bg7188, Bg9640, etc.) [24] also deserve further consideration as key factors in the defense responses to microbial pathogens.

One of the most interesting aspects of AIG genes is their unusual phylogenetic distribution, with molluscs being one of the few groups of lophotrochozoans to possess them. Although no AIG genes were found within the *Octopus bimaculoides* and *Euprymna scolopes* (squid) genomes, more representatives need to be examined to assess presence or absence of AIG genes among cephalopods. The five gastropods and three bivalve genomes available reveal different levels of AIG gene expansion, ranging from 23 to 111 (Table 2). Of the 91 *B. glabrata* AIG genes with complete AIG1 domain, 64 were identified as GIMAPs, with both of these totals being higher than for any of the other nine mollusc species examined. With the exception of *Danio* (zebrafish), *B. glabrata* has more GIMAP loci than any other species thus far investigated.

The AIG gene tree is complex and does not simply recapitulate phylogenetic relationships among the taxa represented. Nonetheless, some phylogenetic signals are provided in the tree. For instance, the AIG genes for three relatively closely related heterobranch gastropods (*A. californica, E. chlorotica* and *B. glabrata*) are grouped together from a single major branch. The terminal branches stemming from this major branch show a remarkable intercalation of expanded subgroups of AIG genes representing all three species. Ten separate subgroups are seen for *B. glabrata*, whereas both *A. californica* and *E. chlorotica* have five. The more phylogenetically distant coenogastropod *Pomacea canaliculata* exhibits a separate expansion of AIG genes stemming from one major branch. How this picture would change with the addition of sequences from other coenogastropod species will prove to be most interesting. AIG gene sequences from the most basal gastropod, *L. gigantea*, represented on the tree occupy two major branches, one unique to *L. gigantea* but not expanded and nesting between the two gastropod branches identified above, and the second nested within a major branch that is otherwise mostly populated by bivalve AIG sequences. This second major branch is once again noteworthy for having smaller intercalated subgroup expansions representing all three bivalve species as well as *L. gigantea*. The extent to which the congeners *C. gigas* and *C. virginica* differ with respect to their AIG gene repertoires is noteworthy. This is quite in contrast to the differences in AIG gene repertoires noted between *B. glabrata* and *B. pfeifferi* in Fig. 8, for which differences are manifested mostly on the terminal branches on the tree. This implies that following a basic expansion in an ancestral species, there has since been only minor modifications as might be expected for two related species estimated to have diverged within the last 5 million years [49]. Additional sampling from more taxa will be required before we can fully interpret the patterns exhibited by the diverse repertoires of AIG genes among molluscs.

Given the status of current data and tool availability, there are caveats and limitation to our study. First, accuracy of AIG gene predictions is limited by completeness and accuracy of the reference genome and its annotation. AIG genes may be underestimated because some scaffolds do not cover a complete gene. Multi-domain AIG genes may have been overlooked if their component domains were on different scaffolds. Similar issues arise with transcriptome assembly if AIG expression is limited to particular organs and conditions. Genes not identified within a transcriptome are not necessarily absent from, or always silent within, the genome. Similarly, genome annotation is limited by our current knowledge of gene models and RNA-Seq/EST data. Second, our study used as a starting point the nine annotated molluscan genomes, so it skipped the steps involved in the initial annotation process. Gene annotation efforts may not always be able to fully distinguish between protein coding, or non-coding genes or pseudogenes. Further wet lab molecular techniques will be required for the final confirmation of existence and functionality of the AIG genes identified here. Third, the HMM profile/models used to scan signatures were built from protein databases containing large number of sequences from model organisms, so are likely to be somewhat biased against reliable domain identification in non-model organisms. This creates some uncertainty with respect to novel domain discovery. For example, some of the *B. glabrata* AIG genes contain long lengths of protein-encoding sequences with no known function. As BLAST searches of such sequences returned nothing from the NCBI non-redundant protein database, they may represent completely novel stretches of functional sequence, or reflect an assembly or annotation error. Fourth, given the complexity of the coiled-coil domain families including some subfamilies that are structurally conserved but have divergent sequences, AIG genes currently categorized as lacking coiled-coils may require reconsideration as bonafide GIMAPs as new prediction tools for coiled-coil domains are developed.

## Conclusions

A systematic investigation of the *B. glabrata* genome has revealed the presence of 91 AIG genes with complete AIG1 domains, more than known for any other organism except zebrafish. Most (64) AIG family members in *B. glabrata* are GIMAPs. The *B. glabrata* AIG1 domain appears in several novel combinations with other domains suggestive of a variety of possible functions. Several tandemly-arrayed AIG gene clusters appear in the *B. glabrata* genome, consistent with a process of segmental gene duplication in their origin. Some *B. glabrata* GIMAPs are strongly upregulated following exposure to bacteria or to *S. mansoni* indicative of a role in immunity. A role in interactions with *S. mansoni* is further suggested by the high constitutive levels of expression shown by several AIG genes from schistosome-resistant BS-90 snails as compared to susceptible M line snails. With respect to their distribution among molluscs, AIG genes are found in the available gastropod and bivalve genomes but thus far appear to be absent in cephalopods. The *B. glabrata* AIG genes localize within one major group on the AIG gene tree that includes only AIG genes from other heterobranch gastropods, but subgroups of *B. glabrata* AIGs are intercalated among the sequences of these other heterobranchs suggesting they have undergone several separate amplifications specific to *B. glabrata.* The curious pattern of apparent ancient presence followed by patterns of apparent loss in some lineages and expansions in others presents a fascinating puzzle awaiting further clarification. The approach taken here will help provide a framework for systematic experimental characterization of new biochemical and biological functions of AIG or GIMAP genes using modern tools like CRISPR/Cas knockout technology [50]. More specifically, we seek to learn how different species and strains of *Biomphalaria* compare with respect to how their AIG genes function in response to infection with the medically relevant parasite, *S. mansoni*.

## Methods

### Data retrieval

Protein and coding regions of gene sequences (CDS) and genome annotation features of *B. glabrata* BB02 strain, BglaB1.6 [36] were downloaded from VectorBase (https://vectorbase.org/organisms/biomphalaria-glabrata/bb02/bglab16) [51]. Nine available and well-annotated molluscan genomes were downloaded from NCBI RefSeq [52] and GenBank [53]: the bivalves *Crassostrea gigas* (GCA_000297895.1), *Crassostrea virginica* (GCA_002022765.4), and *Mizuhopecten yessoensis* (GCA_002113885.2); the gastropods *Aplysia californica* (GCA_000002075.2), *Elysia chlorotica* (GCA_003991915.1), *Lottia gigantea* (GCA_000327385.1), *Pomacea canaliculata* (GCA_003073045.1); and the cephalopod *Octopus bimaculoides* (GCA_001194135.1). The genome and annotation for Hawaiian bobtail squid *Euprymna scolopes* which became available during the course of this study were obtained from CephRes Genomes Database (http://www.cephalopodresearch.org/ceph_gdatab/).

### Computational identification of an AIG gene family in *B. glabrata*

The characteristic AIG1 domain was used for computational analyses in the *B. glabrata* and the nine related molluscan genomes*.* Hmmsearch was performed to search for the AIG1 domain with the HMM model of AIG1 PF04548 from the Pfam database v32.0 [54]. InterProScan 5.34–73.0 software [55] was used to further confirm the AIG1 domain and scan signatures for other functional domains in the AIG1 domain-containing protein sequences of each species. Conservative motifs including the G1-G4, G5/IAN motifs and a conserved box (CB) of the AIG1 domain were scanned within the CDD database [56] using CD-Search tools [57]. Additional features of AIG protein transmembrane domains and topology were predicted with TMHMM 2.0 [58], and coiled-coils were predicted by Coils [59] and their oligomeric state was predicted using LOGICOIL [60].

### Organ specific expression analysis of AIG genes in *B. glabrata* BB02 strain

RNA sequencing reads of 12 organs from *B. glabrata* BB02 strain [36] were downloaded from NCBI Sequence Read Archive (SRA) with SRA run accession numbers SRR1509459- SRR1509470, and SRR1509473. The prefetch and fastq-dump command in the SRA Toolkit (http://ncbi.github.io/sra-tools/ and the SRA Toolkit Development Team) fastq files were used to download SRA data from NCBI, and then to extract fastq format sequences. The raw reads of each tissue were trimmed based on the base calling quality and read length using Trimmomatic v0.36 [61]. Clean reads were mapped to the reference *B. glabrata* BB02 genome using STAR v2.2.1 [62]. Gene counts measured in reads per kilobase of exon per million reads (RPKM) values were calculated using StringTie v1.3.5 [63]. Heatmap figures were generated using the R package pheatmap 1.0.12 [64].

### AIG homolog search and expression analysis of two previous studies on *Biomphalaria spp*

From the microarray study of expression of BS-90 strain *B. glabrata* exposed to bacterial and schistosome-like PAMPs [24], all probes listed in the differential expressed genes were selected. Probe sequences were searched against cDNA sequences of BB02 *B. glabrata* using BLASTn with at least > 95% match identity*.* In the RNA-Seq study on *Biomphalaria pfeifferi* snails exposed to *Schistosoma mansoni* [25], homologs of AIGs from *B. pfeifferi* were identified through searching assembled transcriptome sequences by using BLASTp with cutoffs of identity (> 70%), query coverage (> 90%) and subject coverage (> 90%). For both of these searches, the corresponding gene IDs of AIG homologs for BB02 *B. glabrata* in Vectorbase were identified for further comparison analysis.

### Transcriptomics study on AIG genes in schistosoma susceptible- and resistant-strains of *B. glabrata*

To further investigate AIG genes expression in *B. glabrata*, a comprehensive transcriptomics study of two *B. glabrata* strains (BS-90 snails resistant to *S. mansoni*, and M line snails susceptible to this parasite) was carried out using next generation sequencing. *Schistosoma mansoni* (PR-1 strain) and the two strains of *B. glabrata* were maintained at Biology Department of the University of New Mexico as previously described [65, 66]. Juvenile snails (5-8 mm diameter) of both BS-90 and M line were individually put in the wells of 24-well plates, in 2 ml artificial spring water (ASW) and exposed to ~ 20 *S. mansoni* miracidia per snail, for 6 h. Control snails for each strain were treated similarly but were not exposed to *S. mansoni* miracidia, and represent the snails used to establish constitutive (resting) levels of AIG gene expression.

Snails of each group (Additional file 4: Table S3) were moved to aerated aquaria containing ASW at 25–27 °C and fed with lettuce. Both *S. mansoni*-exposed snails and time-matched unexposed control snails of both strains were taken at 0.5, 2, and 40 days post-exposure (dpe); note that the same unexposed snails were used as controls for both the 2- and 8 dpe exposed snails because snails of these groups did not differ in size. These times were chosen to match key stages of *S. mansoni* development in the snail: 0.5 days, early penetration; 2 days, mother sporocyst establishment; 8 days, daughter sporocyst production; 40 days, full-fledged infection with production (shedding) of cercariae. Snails sampled at 0.5, 2, and 8 dpe were juveniles but snails collected at 40 dpe were adults because unexposed control snails produced eggs (*S. mansoni*-infected snails are typically castrated by infection). For each strain and sampling time, 7–9 snails were collected. At 40 dpe, all remaining snails of both strains were isolated and checked for *S. mansoni* cercariae shedding. For this purpose, snails were placed individually in wells of 12-well plates in 2 mL ASW and held under artificial light for 2 h. As expected, no resistant BS-90 snails shed cercariae. All snails were then allowed to recover in aquaria for 1–2 days prior to sampling. Snails sampled for this study were individually preserved in a 1.7 ml tube containing 1000 μl of TRIzol™ reagent (Invitrogen) and stored at − 80 °C until extraction.

The RNA and DNA extraction of individual snail samples followed TRIzol™ manufacturer’s instructions (Invitrogen). PCR with *S. mansoni* specific primers aimed to the *ND5* gene of *S. mansoni* [67] was used to verify the *S. mansoni* infection status for each schistosome-exposed snail. Only those snails exposed to *S. mansoni* and confirmed with PCR to be positive were considered to be “exposed” snails for this study. RNA samples were further purified using PureLink RNA Mini Kit (Thermo Fisher Scientific) to yield high-quality RNA. Quality and quantity of RNA extracted from each sample were measured with a Nanodrop 2000c spectrophotometer (Thermo Fisher Scientific) and Agilent 2100 Bioanalyzer (Agilent RNA 6000 Pico kit), respectively. RNA samples were stored at − 80 °C and were used for library preparation within one week.

According to the sampling scheme in Additional file 4: Table S3, 3~6 replicates per group per sampling time point were selected for library preparation. Complementary DNA (cDNA) synthesis and Illumina NextSeq 500 sequencing was performed at the Molecular Biology Facility, Biology Department, the University of New Mexico. Synthesis of cDNA from each sample and library preparation followed the KAPA mRNA HyperPrep Kit Illumina® Platforms protocol (Roche). Complementary DNA libraries were paired-end sequenced (2 × 150 base reads) on an Illumina NextSeq 500 instrument (Illumina).

### Differential expression (DE) analysis of the transcriptomics study

For both M line and BS-90 strains, Illumina RNA Seq output for unexposed time-matched controls and PCR-verified *S. mansoni*-exposed snails including raw reads with a score above 20 and length of at least 36 nt, were mapped to the reference BB02 *B. glabrata* genome [36] (Additional file 4: Table S3). Six groups of unexposed snails and 8 groups of *S. mansoni*-exposed snails were considered for various comparisons, including constitutive level comparisons between BS-90 and M line strains. The raw sequence data are available at NCBI under SRA accession: PRJNA591872.

All raw reads from Illumina sequencing were downloaded to local servers for bioinformatics analysis. Raw reads were trimmed and filtered using Trimmomatic v0.36 [61] with slide window of 4 nt, average score above 20 and minimum length of 36 nt. Filtered high quality reads were sorted based on the sample-specific adapters and mapped to the annotation updated *Biomphalaria glabrata* BB02 strain genome [36] from VectorBase [51], using STAR 2.5.3a [62]. Gene expression levels were estimated using software featureCounts [68]. Differential gene expression (DE) analysis was performed by using EBSeq v1.22.0 [69] with normalized clean reads, and DE analysis results were organized using SARTools 1.6.6 [70]. Workflow of read trimming and mapping was build using Unix shell commands with application GNU-Parallel [71] to perform jobs in parallel. A posterior probability of differential expression (PPDE) ≥ 0.95 for EBSeq was set as cutoff for DE analysis. Due to the large number of DE genes discovered, only those DE genes with fold change (FC) value greater than 2 (FC > 2) in either up- or down-regulated were taken into further analyses. For constitutive differences, values for BS-90 snails were compared to baselines from corresponding M line snail groups. Within each snail strain, values for *S. mansoni*-exposed snails were compared to baselines from unexposed control snails time-matched to the same exposed group (Additional file 4: Table S3).

### Phylogenetic and sequence analysis

Based on InterProScan scanning results, AIG1 domain sequences were extracted from protein sequences of *B. glabrata*, and aligned using the accurate L-INS-i method [72] imbedded in the MAFFT’s program [73] through the JABAWS platform [74]. AIG1 domain sequences of *Pomacea canaliculata* (Pc_XP_025083382.1, Pc_XP_025083383.1, and Pc_XP_025111428.1) were included in the analysis as an outgroup. Additional sequences of AIG1 domains from selected eukaryote species, including *Arabidopsis thaliana* (thale cress), *Branchiostoma floridae* (the Florida lancelet), *Danio rerio* (zebrafish), *Homo sapiens* (human), *Physcomitrella patens* (spreading earthmoss), and *Saccoglossus kowalevskii* (acorn worm), were obtained from Additional files 7 & 8 in Weiss et al. (2013). Integrity of AIG1 domains was checked and only ones with all G1-G4, CB and G5/IAN motifs were kept for phylogenetic tree construction. Multiple sequence alignment was visualized and sequences were manually trimmed with the multiple sequence alignment view editor Jalview v2.10.5 [75]. Maximum likelihood phylogeny was inferred by running IQ-TREE v 1.6.10 [76], using the best model search with 1000 bootstrap search replicates, and using standard model selection followed by tree inference. The tree was viewed with FigTree v1.4.4 (http://tree.bio.ed.ac.uk/software/figtree/). Similarly, phylogenetic analysis and visualization was applied to both *B. glabrata* and nine additional molluscan genomes for which AIG1 domains were present, with *Acropora millepora* as an outgroup.

### Other analysis

Signal peptide search was performed using SignalP 4.0 [77]. Table summary and figure generation were performed with the R statistical computing environment [78] and Bioconductor [1], including the following packages: ggplot2 [79], reshape2 [80], magic 2.0 (https://docs.ropensci.org/magick/index.html), ggtree 3.9 [81], and openxlsx v4.1.0.1 [82]. Intermediate data analysis was done with an in-house parallel pipeline facilitated with the GNU Parallel tool [71].

## Supplementary information

**Additional file 1: Figure S1.** Genome-wide locations of AIG footprints in the *B. glabrata* BB02. The evolutionary footprints of AIG genes in *B. glabrata* contain three types: GIMAP (AIG gene with coiled-coil domain), AIG gene without coiled-coil domain, and partial AIG. Scaffold backbones were drawn with gray lines. Scaffolds longer than the figure region were marked with gray dots on left or right end of the gray lines. Genes on the forward strand were marked out using left-to-right arrows above scaffold lines, showing GIMAP genes (blue) and AIG genes (sky blue). Genes on the reverse strand were marked out using right-to-left arrows below scaffold lines, showing GIMAP genes (red) and AIG genes (pink). Partial AIGs (black) were showing on both forward and reverse strand. Scaffold IDs were labeled above each scaffold. Numbers in parenthesis after scaffold IDs are total number of AIG genes (with and without coiled-coils) on the scaffold. Gray parentheses enclosed genes within the same tandem array (no other genes in between).

**Additional file 2: Table S1.** Consensus sequences of motifs within AIG1 domain among selected organisms. G1, G2, G3, CB, G4, G5/IAN are conserved motifs within AIG1 domain. The consensus sequences of these motifs in different organisms may differ or be absent.

**Additional file 3: Table S2.** Features of the predicted AIG footprint in *B. glabrata* The 111 AIG genes (complete and incomplete) and the 148 proteins were detailed, with types of AIG gene, predicted domain architecture, coiled-coil, multimer forms, and genome location.

**Additional file 4: Table S3.** Overview of the 14 snail groups considered and sequencing information for the RNA-Seq study Two strains of *B. glabrata* snails were used for this study: schistosome-resistant BS-90 and -susceptible M line snails were sampled at 0.5, 2-, 8- and 40-day post-exposure to *S. mansoni*. Time-matched unexposed BS-90 and M line were sampled as control for differential expression analysis. Totals of 3~6 biological replicates for each group with raw reads (9–18 million reads/snail sample) were obtained.

**Additional file 5: Table S4.** Summary of AIG gene expression in *Biomphalaria* species from all related studies There are five relevant expression datasets available. I). AIG genes expression in 12 different organs from unstimulated BB02 *B. glabrata*; II). a microarray study of schistosome resistant BS-90 *B. glabrata* injected with different PAMPs, including LPS, PGN, and FCN; III). An RNA-Seq transcriptomics study for field-derived Kenyan *B. pfeifferi* exposed for 1, 3, or 30+ days to *S. mansoni,* relative to unexposed controls; IV). An RNA-Seq study for *S. mansoni*-resistant BS-90 snails relative to susceptible M line snails at constitutive level (no exposure to *S. mansoni*), and V) for both M line and BS-90- snails, intrastrain comparisons of snails exposed for 0.5, 2, 8- and 40-day to *S. mansoni*, relative to time-matched unexposed controls.

**Additional file 6: File S1.** Multiple sequence alignment of AIG1 domains from selected eukaryotes.

## Data Availability

The raw RNA sequencing data are available at NCBI under SRA accession: PRJNA591872. All other data generated or analyzed during this study are included in this published article and its supplementary information files.

## Abbreviations

AIG1: AvrRpt2-induced gene
ASW: Artificial spring water
CARD: Caspase activation and recruitment domain
CB: Conserved box
DD: Death domain
DE: Differential expression
Dpe: Day post-exposure
FCN: Fucoidan
GIMAP: GTPases of immunity associated proteins
IAN: Immune-associated nucleotide-binding proteins
LPS: Lipopolysaccharide
ML: Maximum likelihood
PAMPs: Pathogen associated molecular patterns
PGN: Peptidoglycan
PPDE: Posterior probability of differential expression
TM: Transmembrane domain
TPM: Transcripts per million
