# Supplementary figures and images for: Genome-wide discovery, and computational and transcriptional characterization of an AIG gene family in the freshwater snail Biomphalaria glabrata, a vector for Schistosoma mansoni
Source: BMC Genomics. 2020 Mar 2;21:190. doi: 10.1186/s12864-020-6534-z (PMC7053062; doi:10.1186/s12864-020-6534-z)

# Scaffold ID (number of AIG genes and partial AIGs)

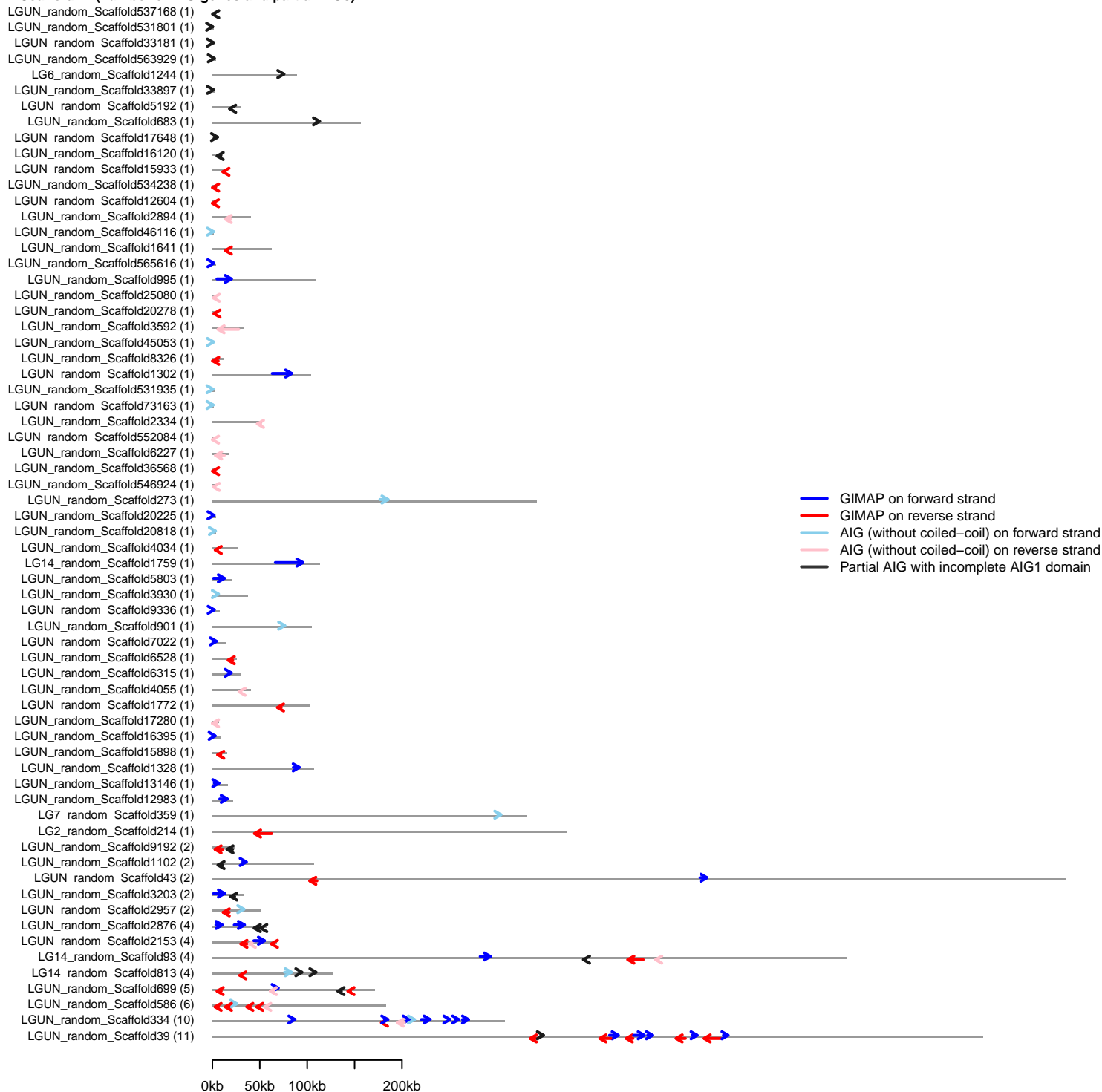

Supplement: Supplementary file 1 — Additional file 1: Figure S1. Genome-wide locations of AIG footprints in the B. glabrata BB02. The evolutionary footprints of AIG genes in B. glabrata contain three types: GIMAP (AIG gene with coiled-coil domain), AIG gene without coiled-coil domain, and partial AIG. Scaffold backbones were drawn with gray lines. Scaffolds longer than the figure region were marked with gray dots on left or right end of the gray lines. Genes on the forward strand were marked out using left-to-right arrows above scaffold lines, showing GIMAP genes (blue) and AIG genes (sky blue). Genes on the reverse strand were marked out using right-to-left arrows below scaffold lines, showing GIMAP genes (red) and AIG genes (pink). Partial AIGs (black) were showing on both forward and reverse strand. Scaffold IDs were labeled above each scaffold. Numbers in parenthesis after scaffold IDs are total number of AIG genes (with and without coiled-coils) on the scaffold. Gray parentheses enclosed genes within the same tandem array (no other genes in between). [file 12864_2020_6534_MOESM1_ESM.pdf]
